# Triggering avalanche-like ultraviolet photomultiplication phenomena in ultrathin amorphous/crystalline gallium nitride heterostructures

**DOI:** 10.1126/sciadv.aea7319

**Published:** 2026-03-11

**Authors:** Dongyang Luo, Haochen Zhang, Yong Yan, Wei Chen, Danhao Wang, Huabin Yu, Xin Liu, Yang Kang, Muhammad Hunain Memon, Zhixiang Gao, Yuanmin Luo, Si Liu, Wengang Gu, Boon S. Ooi, Lan Fu, Sheng Liu, Haiding Sun

**Affiliations:** ^1^ iGaN Laboratory, School of Microelectronics, University of Science and Technology of China, Hefei 230029, P. R. China.; ^2^ Photonics Laboratory, King Abdullah University of Science and Technology, Thuwal, Saudi Arabia.; ^3^ Research School of Physics, Australian National University, Canberra, ACT 2600, Australia.; ^4^ School of Integrated Circuit, Wuhan University, Wuhan 430072, P. R. China.

## Abstract

Avalanche phenomena, triggered by carrier multiplication through impact ionization under high electric fields, form the operating principle of avalanche photodiodes. However, this process inevitably leads to amplified dark current, thereby limiting the photo-to-dark-current ratio despite large gain. Here, we demonstrate avalanche-like nonlinear photocurrent amplification while we maintain ultralow dark current in an amorphous-gallium oxynitride (a-GaON)/gallium nitride (GaN) heterostructure formed via a two-step “amorphization-recrystallization” process. Instead of relying on the impact ionization process, the engineered amorphous/crystalline interface enables trap-assisted photo-induced carrier multiplication behavior at low voltages, yielding a large gain (3.9 × 10^6^) and ultrahigh responsivity (4.3 × 10^7^ amperes per watt) at 35 volts, with an ultralow dark current (~0.7 picoamperes), which are strongly competitive relative to state-of-the-art ultraviolet avalanche photodiodes. We further present proof-of-concept ultraviolet hardware systems incorporated with the newly constructed device. This amorphous/crystalline interfacial engineering strategy presents an unexploited, simple, and scalable device paradigm for fabricating advanced photodetectors in next-generation compact and integrated optoelectronic systems.

## Teaser

Giant photomultiplication with ultralow dark current achieved in amorphous-GaON/GaN heterostructures.

## INTRODUCTION

The avalanche or carrier multiplication effect, typically triggered by impact ionization processes in semiconductors (*1*, *2*), plays a crucial role in the construction of electronics (*3*, *4*) (e.g., avalanche diodes) and optoelectronics (*5*, *6*) [e.g., avalanche photodiodes (APDs)]. When an APD is operated in Geiger mode at a bias above the breakdown voltage, a steeply nonlinear and self-sustaining avalanche phenomenon occurs in response to the absorption of very low optical power, promising greater gain and higher responsivity than other types of photodetectors (*7*–*9*). Thus, APDs act as dominant components in optoelectronic systems that are required to detect relatively weak light, typically for long-distance sensing (*10*), optical communication (*11*), and even quantum applications (*12*). To date, not only conventional Si-, Ge-, or GaAs-based APDs (*13*, *14*) but also new device architectures based on the emerging low-dimensional materials and heterostructures (*15*, *16*) have demonstrated superior avalanche characteristics. When it comes to the ultraviolet wavelength range, many APDs based on wide-bandgap semiconductors, including Ga_2_O_3_, AlGaN, and SiC have been recently reported (*9*, *17*–*19*). These APDs have shown promising multiplication gain and detectivity metric, but they often rely on relatively complex epitaxial engineering and elaborate device structure designs to achieve avalanche phenomenon while still suffering from relatively high operating voltages with remaining trade-offs between gain and dark current, which would inevitably increase the power consumption, system integration level, and circuit design complexity. Thus, for the sensitive low optical signal photodetection particularly in ultraviolet wavelength, photomultiplier tube (PMT) (*20*) remains as the dominant technology. However, PMTs are typically bulky, fragile, and require high-voltage operation (e.g., >1 kV). For the development of future high-performance integrated and portable optoelectronics with low energy consumption operation (*21*–*24*), an alternative device architecture with performance metrics comparable, if not better, than commercial PMT technology is urgently needed.

Here, we report an avalanche-like nonlinear photomultiplication phenomenon in an ultrathin amorphous/crystalline gallium nitride (GaN) semiconductor heterostructure. We first convert the bulk GaN surface into an ultrathin (sub-5-nm thick) amorphous gallium oxynitride (a-GaON) layer via an in situ two-step “amorphization-recrystallization” process, involving a simple plasma-induced self-amorphization followed by subsequent annealing to tailor the physicochemical properties of the amorphous/crystalline interfaces. The responsivity of the a-GaON/GaN–based device increases by 10^4^ times compared with that of the device fabricated with the pristine GaN film due to trap-assisted charge injection (TA-CI) under strong light illumination, achieving a high responsivity of 6.2 × 10^4^ A/W with a photo-to-dark current ratio of 5 × 10^11^ at 10 V. The a-GaON/GaN–based device notably demonstrates a sudden photocurrent amplification under weak light illumination at an elevated voltage of 35 V, exhibiting a high gain (3.9 × 10^6^) and responsivity (4.3 × 10^7^ A/W), with an extremely low dark current of only 0.7 pA. Unlike the impact ionization–induced avalanche process in conventional APDs, the nonlinear photocurrent increase observed here arises from a trap-assisted space charge–limited current (SCLC) conduction at the a-GaON/GaN heterointerface, which offers an alternative pathway for achieving high gain and low dark current simultaneously. To demonstrate their potential applications, we further implement the device and its array in weak ultraviolet light visualization and imaging systems. Essentially, by using the facile III-V–based amorphous/crystalline heterostructures, our work presents an unexploited, simple, and scalable device architecture promising for future advanced optoelectronic systems.

## RESULTS

### Formation and characterization of the a-GaON/GaN heterostructure

**Fig. 1.**
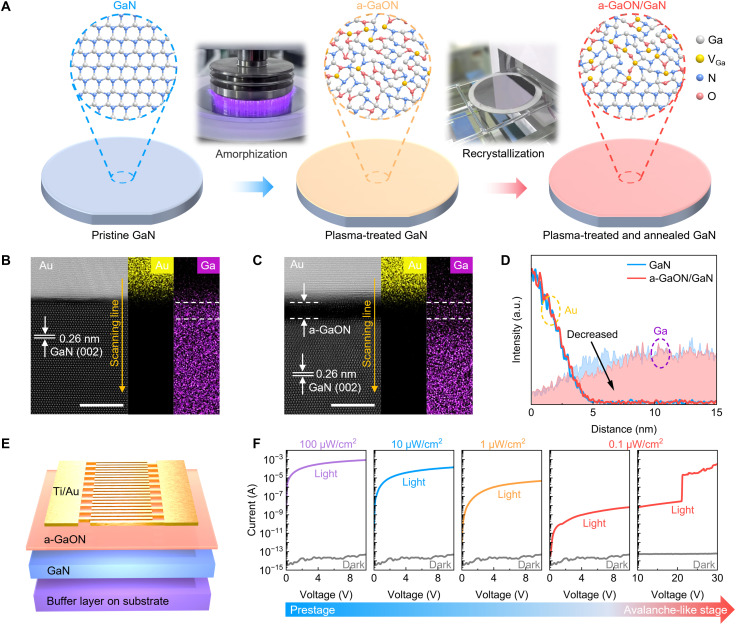
Formation and characterization of the a-GaON/GaN heterostructure. (**A**) Schematic of the two-step process for forming a-GaON/GaN heterostructure. (**B** and **C**) STEM images (scale bars, 5 nm) and corresponding EDS elemental mapping images of (B) pristine GaN and (C) a-GaON/GaN heterostructure samples. (**D**) Line profile of the elemental distribution from EDS mapping. (**E**) Schematic structure of the fabricated a-GaON/GaN device. (**F**) Current versus voltage curves of the a-GaON/GaN device under dark conditions and different light intensities. As the light intensity decreases and the bias voltage increases, the operation mode of the device can transit from the prestage where the photocurrent increases slowly with voltage, to the avalanche-like stage where the photocurrent increases abruptly when the voltage reaches a tipping point. a.u., arbitrary units.

Conventional III-V semiconductor device technology typically prefers single-crystalline structures for subsequent device fabrication to enable effective and efficient carrier generation, migration, and transport. Alternatively, we at least meticulously remove those spontaneously formed amorphous layers on the crystal surface as a routine fabrication process in metallization or material deposition to achieve optimal solid-state device performance (*25*, *26*). However, this mindset might overlook the great potential of amorphous/crystalline heterointerfaces for better device performance after simple structural renovation. Inspired by the success of spontaneously formed SiO_2_/Si amorphous/crystalline heterostructures for the fabrication of modern electronics (*27*, *28*), we attempt a similar self-amorphization process to address challenges facing devices made of III-V compound semiconductors. As a proof-of-concept demonstration, we facilitate the spontaneous formation of an ultrathin a-GaON layer on top of GaN surfaces to form a-GaON/GaN heterostructure. The a-GaON nanolayer is formed on the surface of a GaN-on-Si sample (fig. S1) through a two-step amorphization-recrystallization process, as depicted in Fig. 1A. This process involves plasma treatment using a dielectric barrier discharge (DBD) instrument in an atmospheric environment, followed by high-temperature annealing in an argon atmosphere. The detailed material synthesis conditions are provided in the Materials and Methods. The plasma treatment could transform the surface structure and induce surface amorphization of the pristine GaN film, which can be confirmed via the disappearance of atomic steps on the surface of the pristine GaN sample after plasma treatment, as characterized via atomic force microscopy (AFM) (fig. S2). Direct plasma bombardment on a surface can also generate a substantial number of energetically unfavorable defects (*26*) (e.g., Frenkel pairs and interstitial atoms), which may adversely affect the performance of subsequently fabricated devices. Therefore, a high-temperature annealing process is immediately performed to partially recover the crystalline structure of the GaN surface by promoting recrystallization, eventually resulting in a structurally and thermodynamically stable amorphous/crystalline GaN interface. The scanning transmission electron microscopy (STEM) image of the pristine GaN sample (Fig. 1B) shows lattices, with an average distance between two Ga planes of 0.26 nm, corresponding to the (002) facet of the single-crystalline GaN film (*29*). While after plasma treatment of the pristine sample followed by an optimized annealing process, a sharp and orderly aligned a-GaON/GaN interface is formed, revealing the presence of a ~2-nm amorphous GaON layer on the GaN surface (Fig. 1C). The corresponding energy-dispersive x-ray spectroscopy (EDS) mapping results show that the Ga concentration on the surface of the a-GaON/GaN heterostructure is lower than that on the surface of the pristine GaN sample. Further line profile analysis of the EDS elemental distribution reveals a notable reduction in the Ga concentration within a few nanometers of the a-GaON/GaN interface compared with that of the pristine GaN surface, as shown in Fig. 1D. This might suggest the formation of gallium vacancies in the a-GaON nanolayer.

To confirm this, time-of-flight secondary ion mass spectrometry (TOF-SIMS) and x-ray photoelectron spectroscopy (XPS) are used to analyze the composition and bond state changes in the a-GaON/GaN heterostructure comprehensively. First, TOF-SIMS is performed to analyze the local composition of the a-GaON nanolayer, as shown in fig. S3. Compared with the pristine GaN sample, the a-GaON/GaN sample shows a decreased Ga concentration, which is consistent with the EDS mapping result, along with an increased O concentration at the surface. Next, as shown in fig. S4, the Ga 3d core level spectra for the a-GaON/GaN sample shift toward higher binding energies than those of the pristine GaN sample, indicating an increase in the Ga─O bond content (*30*). These results suggest that plasma treatment and annealing notably alter the surface composition, which could facilitate the formation of an oxygen-concentrated and gallium-depleted GaON nanolayer. Previous studies have reported that Ga vacancies (*V*_Ga_) act as deep acceptors in GaN (*31*). Compared with isolated *V*_Ga_, *V*_Ga_ complexed with oxygen impurities has a lower formation energy and enhanced thermal stability, with the optical transition levels of both types of defects within the bandgap being quite similar (*32*). Consequently, the increase in oxygen impurities promotes the formation of *V*_Ga_, which could be caused by Ga sputtering during plasma treatment (*33*) and out-diffusion during high-temperature annealing (*34*). To quantitatively analyze the defect states, we performed trap density extraction of the a-GaON/GaN structure using a Mercury capacitance-voltage (*C*-*V*) equipment, with the measurement method calibrated in our previous works (*35*, *36*). From the measured *G*_p_/ω-ω curves, the trap energy level (*E*_T_) was calculated to be 0.51 eV, with a high trap density (*D*_T_) exceeding 10^14^ cm^−2^ eV^−1^, as presented and discussed in fig. S5.

To investigate the impact of the amorphous GaON nanolayer (including *V*_Ga_ defects) on device performance, a group of metal-semiconductor-metal photodetectors with interdigital electrodes is fabricated on the basis of pristine GaN and a-GaON/GaN samples. The detailed fabrication processes are provided in the Materials and Methods. A schematic structure of the a-GaON/GaN photodetector is illustrated in Fig. 1E, while the optical microscopy image and structural parameters of the device are presented in fig. S6. By optimizing the annealing temperature, we achieve a substantial photomultiplication gain in the a-GaON/GaN device with a remarkably reduced dark current compared with non-annealed devices (fig. S7). In addition, the XPS results of samples annealed at different temperatures are presented in fig. S8, showing a gradual decrease in Ga─O bond content with increasing temperature, which contributes to the reduction of the dark current.

We further characterize the photoresponse characteristics of the a-GaON/GaN device, as shown in Fig. 1F. At low bias voltages (<10 V), the photocurrent decreases as the light intensity decreases. Under weak light illumination (0.1 μW/cm^2^) at high bias voltages (>20 V), the device exhibits an amplified increase in the photocurrent. When the voltage reaches ~21 V, the photocurrent increases abruptly by more than three orders of magnitude and continues to increase rapidly with increasing applied voltage, exhibiting an avalanche-like nonlinear photomultiplication behavior. Here, the term “avalanche-like” is used to describe the abrupt and nonlinear increase phenomenon of photocurrent in a comprehensible manner, rather than to imply an impact ionization process as in conventional APDs.

We define the stage where no abrupt photocurrent change occurs as the prestage (at relatively high light intensities and low bias voltages) and the stage when the photocurrent exhibits an abrupt increase as the avalanche-like stage (at weak light intensities and higher voltages). The device always exhibits high photocurrent gain in both stages but follows distinctly different mechanisms. The detailed device characteristics and underlying mechanisms are discussed in subsequent sections.

### Photoresponse characteristics of the a-GaON/GaN device in the prestage

**Fig. 2.**
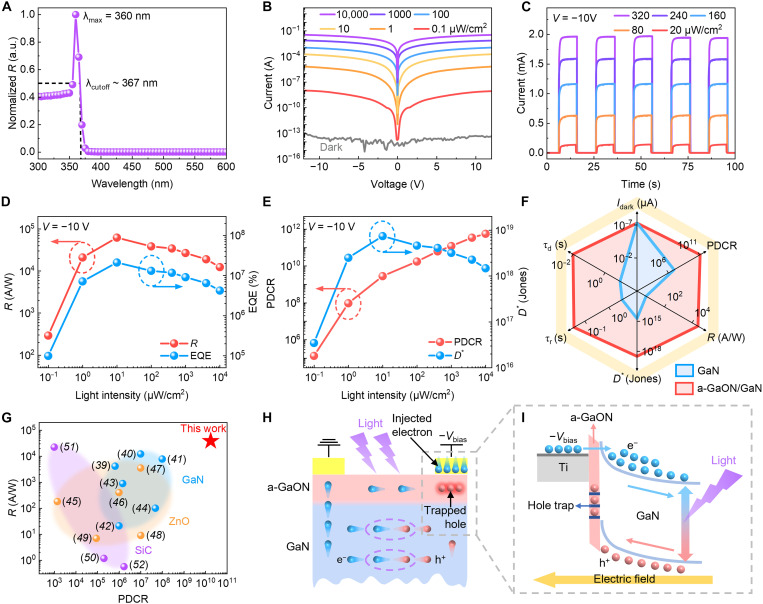
Photomultiplication in the a-GaON/GaN device enabled by TA-CI conduction. (**A**) Normalized spectral responsivity of the a-GaON/GaN device. (**B** and **C**) (B) Static and (C) dynamic photoresponses of the a-GaON/GaN device under 365-nm illumination with different light power intensities. (**D** and **E**) Dependence of (D) *R* and EQE and (E) PDCR and D* of the a-GaON/GaN device on different light intensities. (**F**) Radar plot showing the comparison of *I*_dark_, PDCR, *R*, D*, τ_r_, and τ_d_ for pristine GaN and a-GaON/GaN devices. (**G**) The photoresponse performance of the a-GaON/GaN device, including the *R* and PDCR outperform the previously reported two-terminal device operating in the ultraviolet band. (**H** and **I**) (H) Schematic illustration and (I) band diagram of photomultiplication in the a-GaON/GaN heterostructure under intense light illumination.

According to the above analysis, the a-GaON/GaN photodetector exhibits two distinct photomultiplication phenomena, namely, the prestage and avalanche-like stage. First, we systematically investigate the photoresponse characteristics of the device in the prestage. Figure 2A presents the normalized spectral characteristics of the a-GaON/GaN device, revealing a peak response wavelength at 360 nm and a cutoff wavelength at 367 nm. In addition, the ultraviolet-to-visible rejection ratio exceeds 10^7^, as shown in fig. S9, demonstrating highly selective detection of ultraviolet light. Figure 2B shows the *I*-*V* curves of the a-GaON/GaN photodetector measured in the dark and under 365-nm illumination with different light power intensities (from 0.1 μW/cm^2^ to 10 mW/cm^2^, with each step increasing tenfold), revealing an ultralow dark current (*I*_dark_) of ~50 fA at −10 V and an ultrahigh photocurrent (*I*_photo_). Figure 2C displays the *I*-*T* curves measured under 365-nm illumination with various light power intensities (from 20 to 240 μW/cm^2^) to confirm the repeatability and rapid response speed of the device. The rise time and decay time are extracted in fig. S10. The rise time (τ_r_) and decay time (τ_d_) are ~35 and 10 ms, respectively, defined by the time interval between 10 and 90% of the peak value.

To evaluate the sensitivity of the device under different light intensities, its figures of merit are extracted (*37*). As shown in Fig. 2 (D and E), the device exhibits outstanding responsivity (*R*), external quantum efficiency (EQE), photo-to-dark current ratio (PDCR), and specific detectivity (D*). These parameters are defined as *R* = (*I*_photo_ − *I*_dark_)/(*P*_λ_*S*), EQE = *R*(*hc*/*q*λ), PDCR = (*I*_photo_ − *I*_dark_)/*I*_dark_, and D* = *RS*^1/2^/(2*qI*_dark_)^1/2^, where λ is the wavelength of the incident light, *P*_λ_ is the light intensity, *q* is the quantity of electric charge, *S* is the effective illumination area of the device, *h* is the Planck’s constant, and *c* is the light velocity (*38*). The *R* and EQE can reach maximum values of 6.2 × 10^4^ A/W and 2.1 × 10^7^%, respectively, under 10-μW/cm^2^ light illumination at a bias of −10 V. The ultralow dark current and ultrahigh responsivity result in a superior PDCR and D* of up to 2.8 × 10^9^ and 7.4 × 10^18^ Jones, respectively, under 10-μW/cm^2^ light illumination, suggesting excellent photodetection performance of the device. Given the excellent photoresponse behavior of the a-GaON/GaN device, a systematic comparison of its performance with that of a pristine GaN photodetector and other two-terminal photodetectors operating in the same ultraviolet band is conducted. The a-GaON/GaN device exhibits a substantial photomultiplication gain of up to 10^4^ times that of the pristine GaN device, accompanied by faster rise and decay times, highlighting the advantages of the a-GaON/GaN heterointerface for photosensing purposes (fig. S11). Figure 2F shows the key parameters for our fabricated devices, including *I*_dark_, PDCR, *R*, D*, τ_r_, and τ_d_. The a-GaON/GaN devices maintain an ultralow dark current at the femtoampere level, similar to the pristine GaN devices, but present more than 2 × 10^4^ times higher values of *R*, D*, and PDCR while having a reduced τ_r_ and τ_d_ by factors of 101 and 897, respectively. The details of the pristine GaN device performance are provided in fig. S12. Notably, among previously reported two-terminal photodetectors based on wide-bandgap semiconductors, including GaN (*39*–*44*), ZnO (*45*–*49*), and SiC (*50*–*52*), the a-GaON/GaN device presents notable advantages in terms of key performance metrics, particularly *R* and PDCR values, with the primary data presented and summarized in table S1. Therefore, our devices demonstrate comparable or superior performance to previously reported two-terminal devices based on wide-bandgap semiconductors, as shown in Fig. 2G.

The underlying mechanism of the photomultiplication behavior in the prestage can be attributed to TA-CI in the a-GaON/GaN heterostructure, as depicted in Fig. 2H. Specifically, when the device is negatively biased (−*V*_bias_) and under ultraviolet light illumination, excessive photoinduced electron-hole pairs are generated at the space charge region at the Ti/a-GaON/GaN junction. Thereafter, the photoinduced holes drift to the device surface and are trapped by *V*_Ga_ in the a-GaON layer (*53*). As a result, the separation and migration of photoinduced electrons can be more efficient with a suppressed recombination rate, leading to a multiplied photocurrent. Meanwhile, with sufficient holes trapped at the a-GaON/GaN heterointerface, the electron injection barrier at the metal/GaON interface can be lowered, leading to more electron injection into the device’s active region (*54*), as depicted in the band energy diagram (Fig. 2I). In the absence of illumination, the low carrier concentration in the GaN segment and the high Schottky barrier at the metal-semiconductor interface result in a suppressed dark current.

### Photoresponse characteristics of the a-GaON/GaN device in the avalanche-like stage

Building on the trap-assisted charge injection process during the prestage, further reduction in the light intensity along with a continuous increase in the applied voltage triggers a radical increase in the output photocurrent, thereby resulting in avalanche-like photomultiplication behavior in our a-GaON/GaN device (Fig. 1F). This phenomenon leads to enhanced photocurrent gain under weak light conditions, which is essential for applications where ultraviolet light is relatively dim and weak, such as in flame detection or corona discharge monitoring (*55*, *56*). To elucidate the underlying mechanisms of this radical photocurrent increase in the avalanche-like stage, we further tested the photoresponse characteristics of the a-GaON/GaN device at elevated voltages under weak light conditions.

**Fig. 3.**
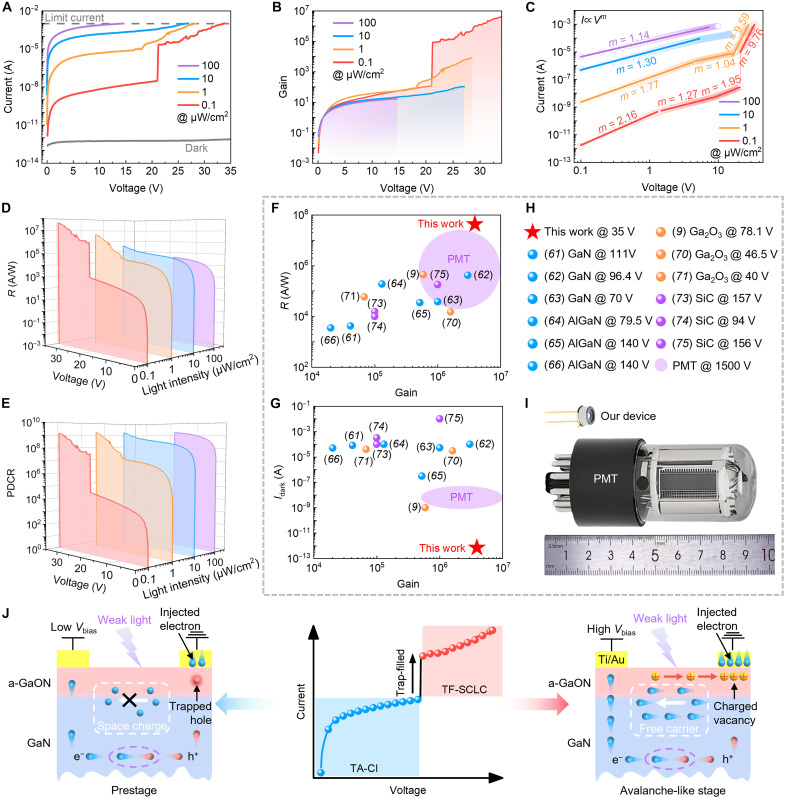
Photomultiplication in the a-GaON/GaN device enabled by TF-SCLC conduction. (**A** and **B**) (A) Current versus voltage curves and (B) gain of the a-GaON/GaN device under 365-nm illumination with different light power intensities. (**C**) Fitted SCLC characteristics at different light intensities. (**D** and **E**) (D) *R* and (E) PDCR as functions of light intensity and applied bias. (**F** and **G**) Performance comparison of the a-GaON/GaN device with previously reported GaN APDs and PMTs, including (F) gain, responsivity, and (G) dark current. (**H**) The corresponding labels of the devices in (F) and (G). (**I**) Schematic comparison of the size and construction between PMT and a-GaON/GaN devices. (**J**) Schematic illustration of the dominant conduction mechanism transitions in the a-GaON/GaN device from TA-CI conduction in the prestage to TF-SCLC conduction in the avalanche-like stage.

Figure 3A shows the current versus voltage curves under 365-nm light illumination at different intensities. To prevent an unrecoverable breakdown, the limit current is set to 1 mA. Notably, the dark current remains exceptionally low even at a bias voltage of 35 V, whereas the photocurrent reaches 1 mA across all the tested light power intensities. The hysteresis window observed in the *I*-*V* curves becomes more pronounced as the light intensity decreases and the bias voltage increases, as shown in fig. S13. This behavior is further highlighted in the dynamic photoresponse of the device under weak light illumination (fig. S14). Under a high applied voltage, the device cannot be effectively turned off when the light source is off. Therefore, we apply a pulsed voltage signal to effectively switch the device on and off. As shown in fig. S15, the device maintains a low dark current (blue line) even with a bias voltage pulse of 35 V under dark conditions. In contrast, under weak light illumination (0.1 μW/cm^2^), the photocurrent increases notably (red line) and can be effectively switched off. The a-GaON/GaN device demonstrates the ability to sustain an extremely low dark current in the absence of light while generating a notably amplified photocurrent under weak light conditions, thereby enabling highly effective detection of weak light signals. The multiplication gain (*M*) is determined via the following relation: *M* = [*I*_photo_(*V*) − *I*_dark_(*V*)]/[*I*_photo_(0) − *I*_dark_(0)] (*9*), where *I*_photo_(*V*), *I*_dark_(*V*), *I*_photo_(0), and *I*_dark_(0) are the multiplied photocurrent, multiplied dark current, unmultiplied photocurrent, and unmultiplied dark current, respectively. Here, the unmultiplied current at 1 V is assigned as the reference for unity gain. As the applied voltage increases, the gain values continue to increase, as shown in Fig. 3B. The gain value also increases as the light power intensity decreases, reaching a value of 3.9 × 10^6^ under a low light intensity of 0.1 μW/cm^2^, demonstrating the excellent weak light detection capability of the device.

To further investigate the underlying principle of photomultiplication phenomena in the avalanche-like stage, we perform a fitting process of the *I*-*V* curves in Fig. 3A. The results show a power law of *I* ∝ *V^m^*, corresponding to a trap-assisted SCLC with an exponential distribution of traps (*57*), as shown in Fig. 3C. Specifically, under high light power intensities of 100 μW/cm^2^, the *m*-index is approximately 1, exhibiting ohmic conduction characteristics (*58*). This is attributed to the reduced electron injection barrier during the TA-CI process. However, under 10-μW/cm^2^ light illumination, the *m*-index reaches 1.3, which deviates from the ideal ohmic contact characteristics, indicating that as the light intensity decreases, the electron injection barrier is less effectively reduced at low light intensities under small bias voltages. At 1 μW/cm^2^, the device has an *m*-index of 1.77 in the first region, but this value decreases to 1.04 in the second region. This suggests that as the bias voltage increases, photogenerated carriers are more effectively generated and transported, leading to more photogenerated holes being trapped by gallium vacancies in the a-GaON layer, which effectively reduces the electron injection barrier. In the third region (*m* = 9.59) at 1 μW/cm^2^, the current increases rapidly, indicating the transition into the trap-filled SCLC (TF-SCLC) regime, where all available trap states are filled by the injected carriers (*59*). Thus, even at a lower light intensity of 0.1 μW/cm^2^, the device exhibits four distinct regions with varying *m*-indices, accompanied by an abrupt current amplification phenomenon. Initially, an *m*-index of 2.16 indicates insufficient barrier lowering under weak light intensity and a small applied voltage. Next, an *m*-index of 1.27 reflects the trapping of more photogenerated holes and a reduced electron-injection barrier at slightly higher voltages. The third region, with an *m*-index of 1.95, corresponds to trap-unfilled SCLC (*59*), following a power law of *I*∝*V*^2^. Last, at the trap-filled limit voltage (*V*_TFL_), the photocurrent undergoes an abrupt change and then transitions into the TF-SCLC regime (*m* = 9.76) (*60*). To validate the role of the a-GaON nanolayer in the TF-SCLC conduction process, a pristine GaN device is also tested as a reference. As shown in fig. S16, the pristine GaN device does not exhibit notable TF-SCLC characteristics under weak light conditions, indicating that the a-GaON layer plays a dominant role in TF-SCLC conduction.

Apparently, the a-GaON/GaN device exhibits high photoresponse performance in the TF-SCLC conduction mode. To further characterize its performance, the *R* and PDCR as functions of light power intensity and applied bias voltage are shown in Fig. 3 (D and E), respectively. The device achieves higher *R* and PDCR under lower light intensities with a larger applied voltage, reaching values of 4.3 × 10^7^ A/W and 1.3 × 10^9^, respectively, under 0.1-μW/cm^2^ light illumination at 35 V. Compared with traditional high-gain wide-bandgap APDs, including GaN (*61*–*63*), AlGaN (*18*, *64*–*69*), Ga_2_O_3_ (*9*, *17*, *70*–*72*), and SiC (*73*–*76*) (data presented in table S2), our a-GaON/GaN device exhibits competitive performance featuring high gain and responsivity while operating at lower bias voltage and through a distinct trap-assisted photomultiplication mechanism, as shown in Fig. 3 (F and G). The corresponding labels for these devices are shown in Fig. 3H. Notably, while conventional GaN APDs multiply both the photocurrent and dark current under high bias voltage, resulting in a PDCR typically less than 1, our a-GaON/GaN device results in a substantial increase in the photocurrent without a corresponding rise in the dark current under an optimal bias voltage. This behavior further suggests that the a-GaON/GaN device demonstrates superior weak light detection capabilities, which are even comparable to those of commercial PMTs (*77*). A schematic comparison of the a-GaON device and PMT in terms of size and structure is shown in Fig. 3I. The a-GaON/GaN device features a low operating voltage with an extremely small dark current, with *R* and gain comparable to those of commercial PMTs, while offering a more compact device structure, making it a promising device for weak light detection in miniaturized system applications.

As discussed above, the TF-SCLC conduction mechanism in the avalanche-like stage notably enhances the photocurrent gain under low light intensities. The working mechanism transition between the prestage and avalanche-like stage is illustrated in Fig. 3J. At a low bias voltage in the prestage, the minimal number of photogenerated carriers in the GaN film under weak light results in fewer photogenerated holes being trapped in the a-GaON layer, leading to an inefficient TA-CI process. Consequently, the conductivity remains low, and most injected electrons are either trapped or forming space charges, hindering carrier transport. In contrast, at high bias voltages in the avalanche-like stage, photogenerated carriers can be more effectively generated and transported. The charged vacancies are activated to migrate under a high electric field, accumulating at the metal/a-GaON interface while effectively reducing the electron injection barrier. As a result, when the traps in the a-GaON nanolayer are filled, many injected carriers can migrate through the heterostructure, leading to a radical increase in the trap-filled space charge limited photocurrent.

### Applications of the devices in ultraviolet visualization and imaging systems

The device operates in TA-CI conduction mode in the prestage, offering reasonable gain under high light intensities. While under weak light, the TF-SCLC conduction mode offers notably greater gain after triggering avalanche-like photomultiplication. By adjusting the bias voltage to switch between TA-CI conduction and TF-SCLC conduction modes, the device effectively detects light intensities across a wide dynamic range. For large-scale fabrication and practical applications, high device uniformity and operational stability are crucial to ensure consistent performance and reliable operation. We therefore evaluated these two aspects. The devices exhibit excellent uniformity, with coefficients of variation of 6.74 and 7.52% under the TA-CI and TF-SCLC modes, respectively (figs. S17 and S18). Furthermore, stability measurements show no noticeable performance degradation in both modes after thousands of operating cycles, confirming the reliable photoresponse characteristics of the device (figs. S19 and S20).

**Fig. 4.**
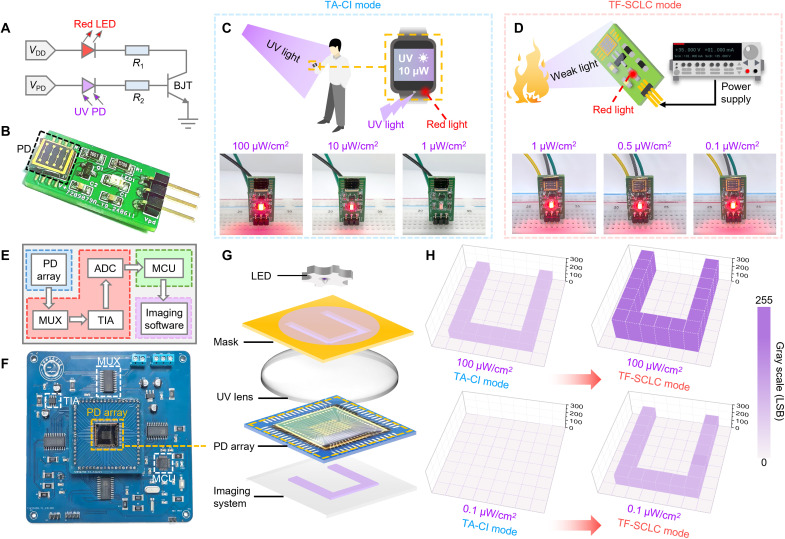
Applications of the a-GaON/GaN devices in ultraviolet visualization and imaging systems. (**A** and **B**) (A) Circuit diagram and (B) photograph of the ultraviolet visualization system PCB. (**C** and **D**) Test results of the ultraviolet visualization system under 365-nm light illumination with different light intensities in (C) TA-CI mode and (D) TF-SCLC mode and their corresponding application scenarios. (**E** and **F**) (E) Architecture diagram and (F) photograph of the imaging system PCB. (**G**) Schematic diagram of the whole imaging system. (**H**) Imaging results of the letter U in TA-CI mode and TF-SCLC mode. UV, ultraviolet; PD, photodetector; MCU, microcontroller unit; MUX, multiplexer; TIA, transimpedance amplifier; ADC, analog-to-digital converter; LSB, least significant bit.

Leveraging the outstanding photoresponse performance, high uniformity, and high operational stability of the a-GaON/GaN devices, we further demonstrate their potential for applications in ultraviolet visualization and imaging systems (*78*–*80*). The ultraviolet visualization system is constructed on a customized printed circuit board (PCB). The circuit diagram and photograph of this system are shown in Fig. 4 (A and B), respectively, with the test platform of this system shown in fig. S21. In our design of the visualization system, a bipolar junction transistor (BJT) amplifier with a gain of ~100 is used to convert the ultraviolet-induced output signal from the a-GaON/GaN device into the driving current to enlighten a red light-emitting diode (LED). Therefore, the detector and a series resistor (*R*_2_) are connected to the gate terminal of the transistor amplifier, and the red LED is connected in series with a protection resistor (*R*_1_) as the output load. When an ultraviolet signal is detected, the a-GaON/GaN device generates a high photocurrent to turn on the BJT and thereby enlightens the red LED.

To validate both the TA-CI and TF-SCLC conduction modes of the device, the visualization system is tested under different bias voltages and ultraviolet light intensities. Specifically, for scenarios requiring visualization of intense ultraviolet radiation, the device can operate in TA-CI mode at a low bias voltage (5 V) and effectively visualize high-level ultraviolet light, indicating the potential of this system in smartwatches for effective ultraviolet exposure early warnings (Fig. 4C) (*81*–*83*). In addition, by operating the device in TF-SCLC mode at a high bias voltage (35 V), the system can still detect low-level ultraviolet light, which is required in scenarios such as flame or corona discharge detection (Fig. 4D). Notably, the photosensitive area of the device in the system is ~0.08 mm^2^. By increasing the photosensitive area and/or reducing the interdigital electrode spacing of the photodetector, this system could effectively detect even weaker light.

The successful demonstration of the ultraviolet visualization system highlights the typical application of a single photodetector. Owing to the uniform complementary metal-oxide semiconductor (CMOS)–compatible plasma treatment process across the entire 6-inch (15.24 cm) GaN epilayer, the development of an imaging system based on a photodetector array on a wafer scale is possible. Here, we develop an integrated PCB subsystem using an 8 × 8 photodetector array. The system architecture and photograph of the PCB subsystem are shown in Fig. 4 (E and F), respectively. This PCB subsystem consists of an 8 × 8 photodetector array, an array data readout circuit hardware module, and a display software module (*84*). Detailed information about the PCB subsystem is provided in Materials and Methods.

Last, the whole imaging system is configured as illustrated in Fig. 4G (a photograph of the whole system is shown in fig. S22). A photomask is used to apply the patterned optical signal generated by an ultraviolet LED, and the ultraviolet lens focuses the optical image onto the photodetector array. Afterward, the PCB subsystem collects data from each pixel and displays the final image on a laptop. Before imaging measurements, the uniformity of all the pixels in the photodetector array is verified to ensure the accuracy and reliability of the imaging system. Initial tests are conducted on all the pixels of the device under dark and illuminated conditions, as depicted in fig. S23, confirming the good uniformity of all the pixels. To further demonstrate the array’s imaging capabilities, the performance of the array is evaluated via a photomask pattern of the letter “U” in both TA-CI mode (bias voltage: 5 V) and TF-SCLC mode (bias voltage: 35 V), as shown in Fig. 4H. In TA-CI mode, notable gray-level contrast is observed between pixels illuminated at 100 μW/cm^2^ and those without illumination, resulting in a clear imaging pattern. However, when the light intensity was reduced to 0.1 μW/cm^2^, a distinct imaging pattern could no longer be observed. In contrast, under the TF-SCLC mode with higher photomultiplication gain, clear imaging is then achieved even at 0.1-μW/cm^2^ light illumination, and the clarity of the pattern is further enhanced at 100 μW/cm^2^. Therefore, by adjusting the bias voltage to switch between these two operation modes of our device, the imaging system effectively captures images of targets under varying light intensities (e.g., in tunnels or remote areas with limited lighting conditions), demonstrating its promising potential for next-generation ultraviolet imaging applications.

## DISCUSSION

We report an amorphous/crystalline heterostructure enabled by a CMOS-compatible amorphization-recrystallization process, e.g., a-GaON/GaN heterostructure. The a-GaON/GaN–based device demonstrates strong photomultiplication phenomena under both intense and weak light illumination. Its responsivity increases by 10^4^ times compared with that of the device fabricated with the pristine GaN film because of TA-CI under intense light illumination. With the continued increase in the bias applied to the device, exceptional abrupt photocurrent amplification can be observed even at lower light exposures, which is attributed to the triggered TF-SCLC conduction mode. A large gain, high responsivity, and low dark current can subsequently be achieved. This transition between conduction modes—controlled by the bias voltage—enables the device to detect light effectively across a wide dynamic intensity range, demonstrating its versatility under various illumination conditions. By leveraging the outstanding photoresponse performance of the a-GaON/GaN heterostructure, we further demonstrate its potential in ultraviolet visualization and integrated imaging systems. This work presents an unexploited architecture of amorphous/crystalline heterostructure design for advanced and highly sensitive photodetectors operating in the ultraviolet band for future portable and miniaturized optoelectronic systems.

## MATERIALS AND METHODS

### Material preparation

The unintentionally doped GaN film was grown via metal-organic chemical vapor deposition on the AMEC Prismo PD5 platform. A 6-inch (15.24 cm) 1000-μm-thick (111)-oriented Si wafer with a resistivity of 0.1 Ω·cm was used as the substrate. First, high-temperature H_2_ cleaning and Al predose were used to prevent Ga meltback. Next, a 200-nm AlN nucleation layer was grown, followed by ~4.5-μm AlGaN and GaN buffer layers, which were both doped with carbon of ~1 × 10^19^ cm^−3^ to form a high-resistance template. Last, 300-nm-thick unintentionally doped GaN was grown at a high temperature of 1100°C to avoid background doping.

An amorphous GaON layer formed on the GaN surface via plasma treatment, which was performed with a DBD instrument (CTP-2000K) at 60 W for 2 min in an atmospheric environment. The samples were subsequently annealed in a furnace (OTF-1200X) at different temperatures (600°, 700°, 800°, and 900°C) for 60 min in an argon atmosphere with a flow rate of 50 standard cubic centimeters per minute.

### Material characterization

The high-angle annular dark-field (HAADF)-STEM image of the sample, which was prepared with a focused ion beam system (Helios NanoLab650), was captured with a JEM-ARM200F. The surface morphology and roughness of the samples were determined via an AFM instrument (Bruker, Dimension Icon) with a tapping-mode conductive probe (TESPA-V2). Monochromatic illumination was provided by a xenon lamp with a monochromator (IHR320, Horiba JY). XPS measurements were conducted with a Thermo Fisher Scientific K-Alpha XPS instrument equipped with an Al Kα source. The binding energy scale of all the measurements was calibrated by referencing C 1s to 284.8 eV. TOF-SIMS measurements were conducted on a CAMECA IMS 7f-Auto instrument, and the sputtering ion source for the TOF-SIMS profile was cesium (Cs).

### Device fabrication and measurements

For the fabrication of the photodetector array, cross-shaped Ti/Au metal stacks (20/50 nm) for subsequent photolithography alignment were first deposited on the pristine sample via electron beam evaporation, and then, a selective plasma treatment was performed with a photoresist mask in the DBD instrument to form the patterned a-GaON. After that, the photoresist was stripped, and the sample was annealed in a tube furnace. Last, Ti/Au metal stacks (20/120 nm) were deposited via electron beam evaporation to form photodetectors, metal interconnects, and wire bonding pads. Each photodetector was based on 11 pairs of interdigital electrodes whose finger length, width, and spacing are 200, 5, and 5 μm, respectively.

The photoresponse performance was tested by a semiconductor parameter analyzer (Agilent B1500A and Agilent B1505A). A 365-nm LED was used as the light source, whose light intensity was calibrated by an optical power meter (Newport model 2936-R). The wavelength-dependent photoresponse was determined via a spectral measurement system (Zolix DSR-OS-X150A-ZKDDZ).

### Ultraviolet visualization and imaging system fabrication

The ultraviolet visualization system consists of a photodetector and a red LED (NCD1206R1) connected with a BJT (MMBT3904), and protection resistors are used to limit the current to prevent device burnout. For the imaging system fabrication, the photodetector array was wire bonded on the top PCB template, and the PCB template was connected to the bottom readout circuit PCB through the pin header and female header connection. The 8 × 8 imaging pixels were connected to four multiplexers (CD74HC4067); thus, all the pixels can be individually addressed by selecting the corresponding input terminal of the multiplexer. Then, the current of each pixel was read out in sequence under the control of a microcontroller unit (MCU; STM32F103C8T6) and converted into a digital voltage signal by a transimpedance amplifier (AD825 and OPA365) and analog-digital conversion in the MCU. The digital signal was further transmitted to a laptop through serial port communication, and customized software was used to show the imaging results on the graphical user interface.
