# Supplementary material for: Triggering avalanche-like ultraviolet photomultiplication phenomena in ultrathin amorphous/crystalline gallium nitride heterostructures
Source: Sci Adv. 2026 Mar 11;12(11):eaea7319. doi: 10.1126/sciadv.aea7319 (PMC12978217; doi:10.1126/sciadv.aea7319)
Supplement: Supplementary file 1 — Figs. S1 to S23 Tables S1 and S2 [file sciadv.aea7319_sm.pdf]

Science Advances
AAAS

Supplementary Materials for

Triggering avalanche-like ultraviolet photomultiplication phenomena in ultrathin amorphous/crystalline gallium nitride heterostructures

Dongyang Luo *et al.*

Corresponding author: Haiding Sun, haiding@ustc.edu.cn

Sci. Adv. **12**, eaea7319 (2026)
DOI: 10.1126/sciadv.aea7319

This PDF file includes:

Figs. S1 to S23
Tables S1 and S2

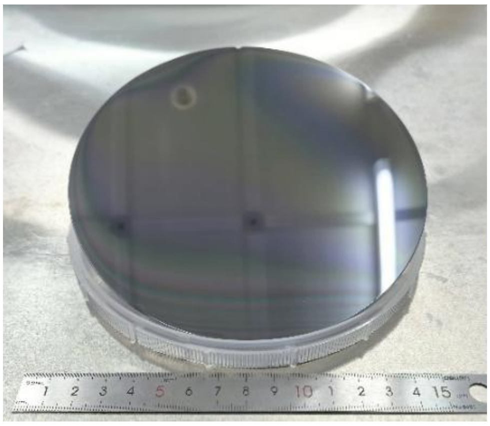

Fig. S1. Photograph of the 6-inch GaN-on-Si wafer.

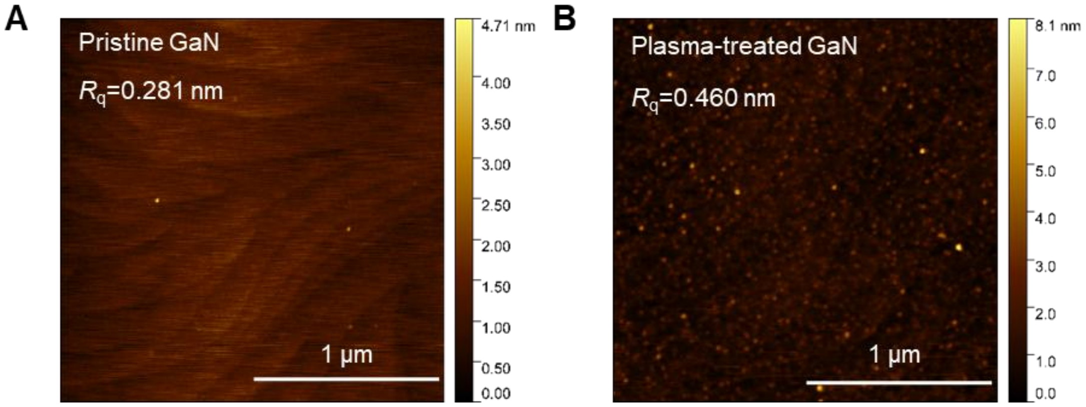

Fig. S2. Film morphology. (**A** and **B**) Atomic force microscopy (AFM)-measured surface morphologies of (A) pristine GaN and (B) plasma-treated GaN. The pristine GaN surface exhibited clear atomic steps with a low root-mean-square surface roughness (R_q) of 0.281 nm. After plasma treatment, R_q slightly increased to 0.46 nm, and the atomic steps disappeared.

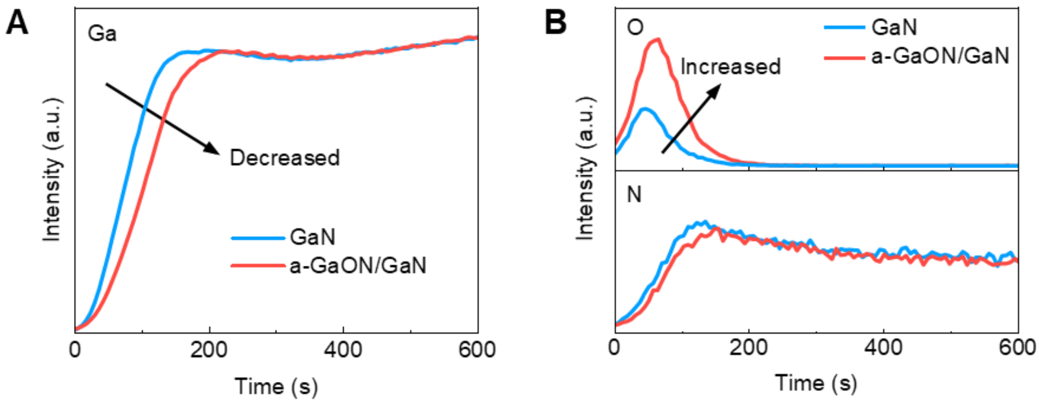

Fig. S3. Time-of-flight secondary ion mass spectrometry results. (**A** and **B**) Time-of-flight secondary ion mass spectrometry (ToF-SIMS) depth profiling results for (A) Ga and (B) O & N in the pristine GaN and the a-GaON/GaN heterostructure samples.

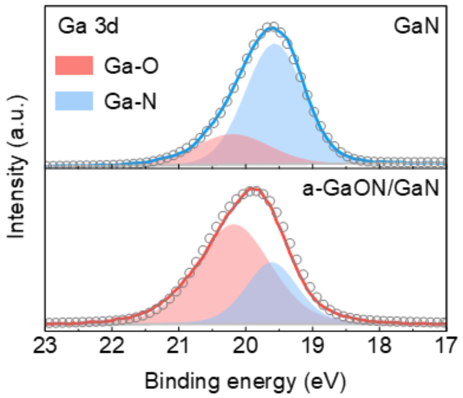

Fig. S4. X-ray photoelectron spectroscopy results. Ga 3d core level spectra of pristine GaN and a-GaON/GaN heterostructure samples.

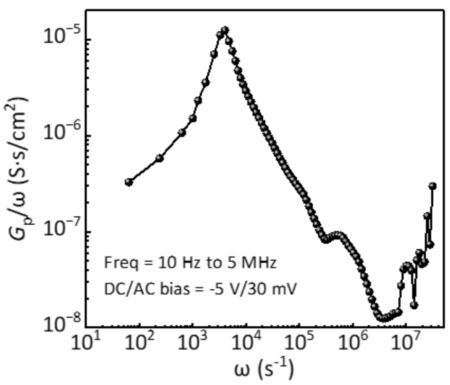

Fig. S5. Trap-density extraction. $G_p/\omega - \omega$ curves of the device under DC/AC bias = –5 V / 30 mV. The testing frequency range was from 10 Hz to 5 MHz, and the DC/AC signal amplitudes were set as –5 V/30 mV. The extraction is based on the following two equations, as given by,

$$\frac{G_p}{\omega} = \ln[1 + (\omega\tau)^2] \times \frac{qD_T}{2\omega\tau} \tag{S1}$$

$$\tau = (v_{th} N_c \delta_T) \exp\left(\frac{E_T}{kT}\right) \tag{S2}$$

where G_p & ω (=2πf) are the measured conductance and testing frequency, q is the elementary charge, D_T is the trap density in (cm^{-2} · eV^{-1}), τ is the trap time constant, v_{th} is the electron thermal velocity as 2.6×10^7 cm/s, N_C is the effective density of charges at the conduction band minimum (CBM) of GaN as $4.3\times10^{14}\times T^{3/2}$ cm^{-3}, σ_T is the capture cross section of trap states as 10^{-14} cm^2, E_T & k & T are the trap energy level in (eV), Boltzmann constant, and Kelvin Temperature in (K). It should be noted that the $(G_p/\omega)_{max}$ occurs at $\omega\tau$ = ~2 according to mathematical property of Eq (S1), producing the corresponding D_T value of ~$2.5(G_p/\omega)_{max}/q$.

The measured $G_p/\omega - \omega$ curves, from which the $(G_p/\omega)_{max}$ value is obtained as 1.24×10^{-5} F/cm^2 at ω = 4014.26 s^{-1}, producing a τ value of 4.98×10^{-4} s and a D_T value of 1.94×10^{14} cm^{-2}·eV^{-1}. Based on Eq (S2), the E_T is calculated as 0.51 eV. These results indicate that at an energy level of 0.51 eV below the CBM, there exist high-density interfacial states over 10^{14} cm^{-2}·eV^{-1} acting as the hole traps.

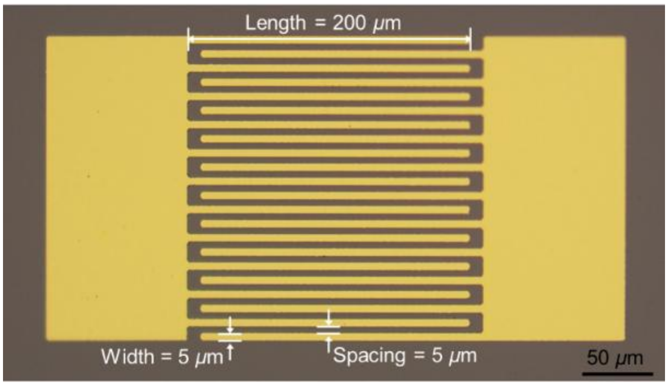

Fig. S6. Device morphology. The optical microscopy of the device (scale bar, 50 μm).

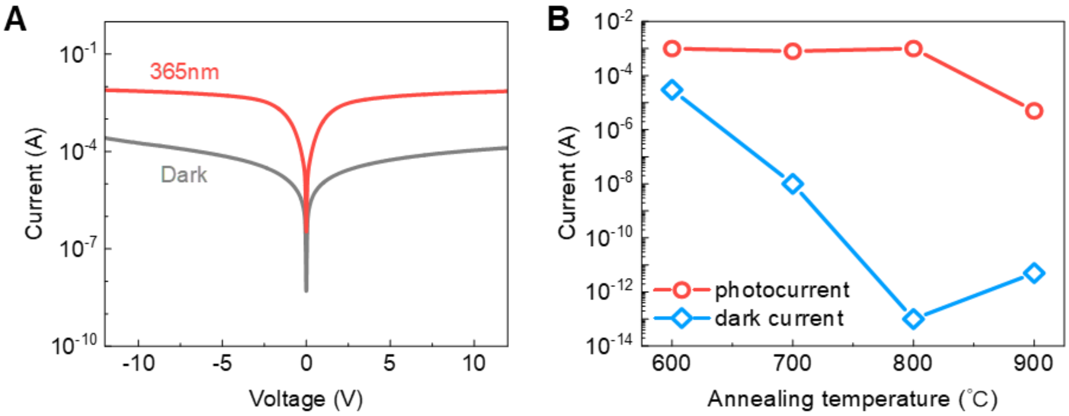

Fig. S7. The influence of annealing temperature on device performance. (**A**) Photocurrent (365 nm illumination with a light intensity of 400 μW/cm^2) and dark current of the plasma-treated sample without the annealing process. Owing to the energetically unfavorable defects introduced by the plasma treatment, the non-annealed device exhibits relatively high dark current. (**B**) Photocurrent and dark current tests were conducted on devices fabricated from plasma-treated samples under different annealing temperatures (at a bias voltage of 5 V and 365 nm illumination with a light intensity of 400 μW/cm^2).

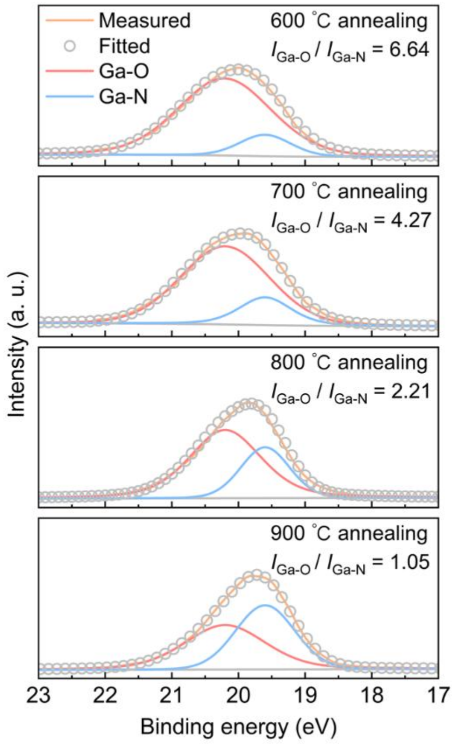

Fig. S8. X-ray photoelectron spectroscopy results. Ga 3d core level spectra of samples after plasma treatment and annealing at different temperatures, with Ga-O bond content decreasing as temperature increases. The reduction in oxygen concentration is attributed to the oxygen out-diffusion, while at 900 °C, possible surface decomposition further accelerates this decrease.

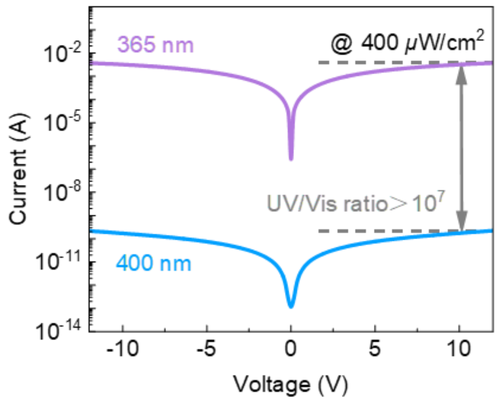

Fig. S9. UV/Vis rejection ratio. Current versus voltage curves of the a-GaON/GaN device under 365 nm and 400 nm light illumination with a power intensity of 400 μW/cm^2.

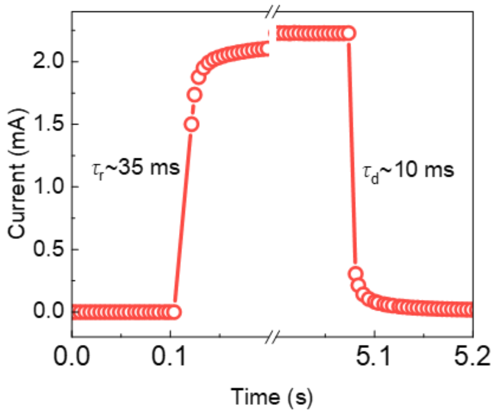

Fig. S10. Photoresponse times extraction. Enlarged rise and decay curves in one period of time-dependent photoresponse curves.

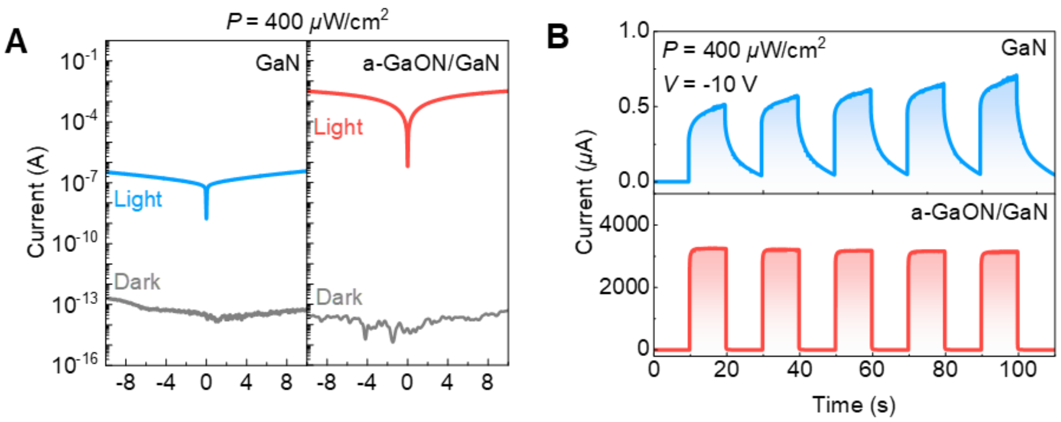

Fig. S11. Comparison of photoresponse performance. (**A** and **B**) (A) Static and (B) dynamic photoresponses of the pristine GaN device and the a-GaON/GaN device under 365 nm illumination**.**

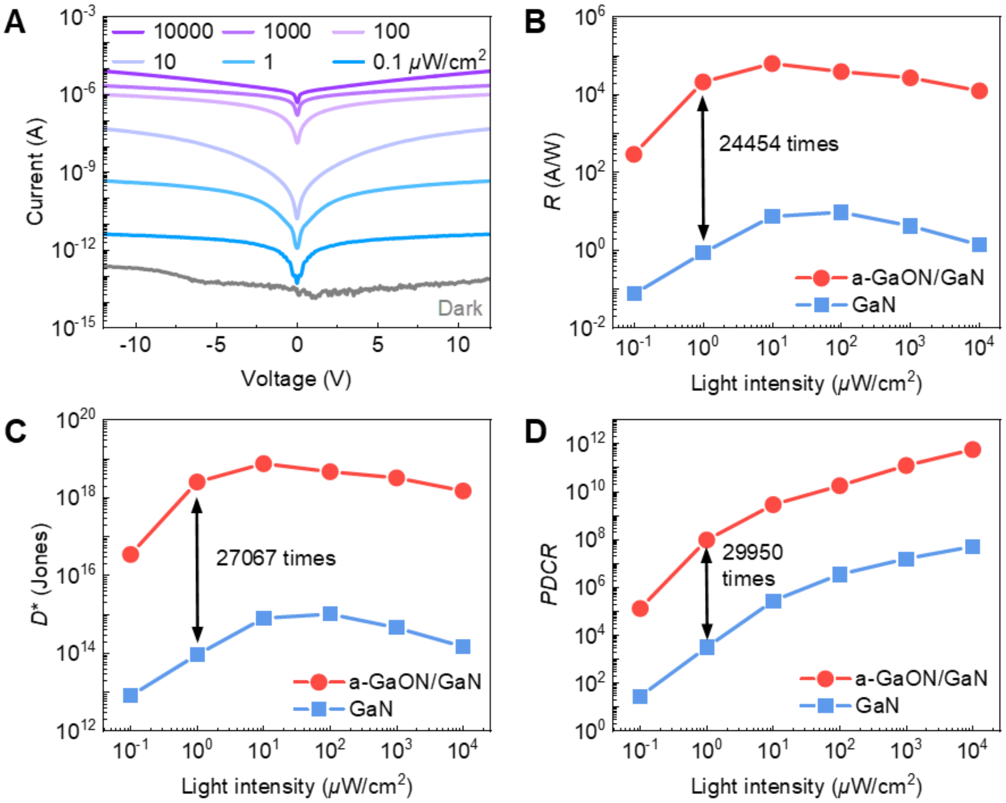

Fig. S12. Pristine GaN photodetector performance. (**A**) Static photoresponse of the pristine GaN photodetector in the dark and under 365 nm illumination with different light power intensities. (**B** to **D**) Dependence of (B) *R*, (C) *D**, and (D) *PDCR* of the pristine GaN device and the a-GaON/GaN device on different light intensities.

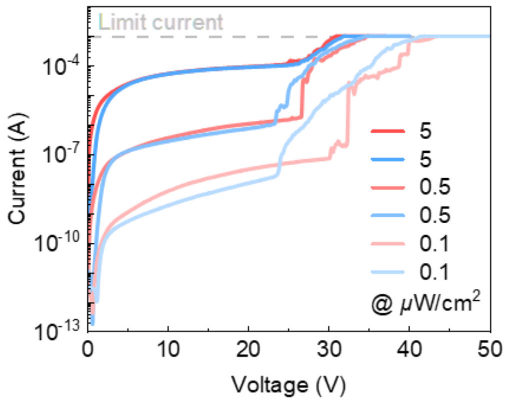

Fig. S13. Current versus voltage curves under different light intensities. Hysteresis curves of the a-GaON/GaN device under different 365 nm light intensities. The red lines correspond to the forward direction sweep, and the blue lines correspond to the backward direction sweep.

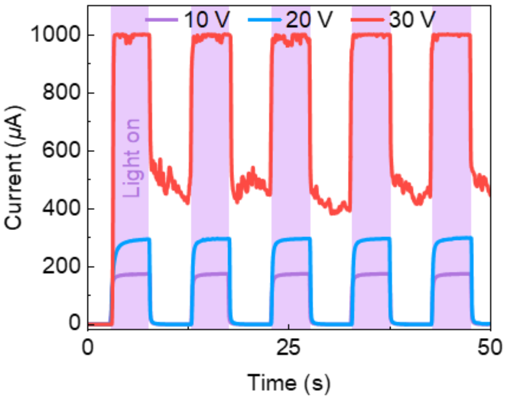

Fig. S14. Time-dependent photoresponse at different bias voltages. Time-dependent photoresponse of the a-GaON/GaN device under 365 nm light with a light power intensity of 10 μW/cm^2 at different bias voltages. When the applied bias voltage reaches 30 V, the device cannot effectively turn off when the light source is switched off.

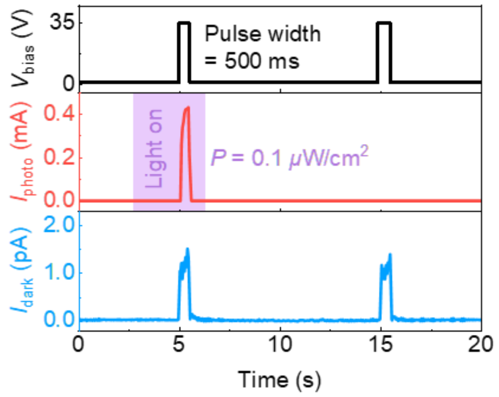

Fig. S15. Dark- and photo-current of the a-GaON/GaN device under pulsed voltage switching. The a-GaON/GaN device maintains a low dark current (blue line) even with 35 V bias voltage pulses (black line) under dark conditions. In contrast, under 0.1 μW/cm² light illumination, the photocurrent increases significantly (red line) and can be effectively switched off. This testing method employs a pulsed voltage to switch the device's avalanche-like mode on and off. For subsequent research, reference can be made to the quenching circuit design of Geiger-mode avalanche photodiodes, which enables automatic shutdown functionality upon photon detection through passive or active quenching mechanisms.

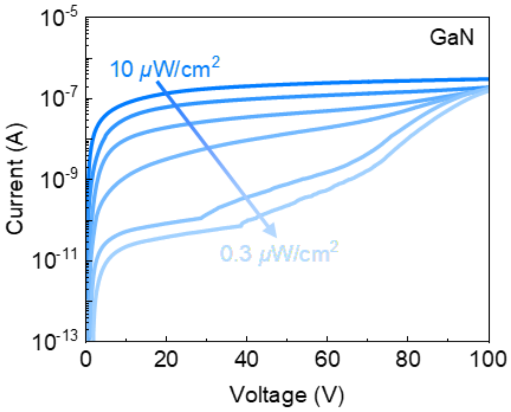

Fig. S16. Current versus voltage curves of the pristine GaN photodetector under low light intensities and high bias voltages.

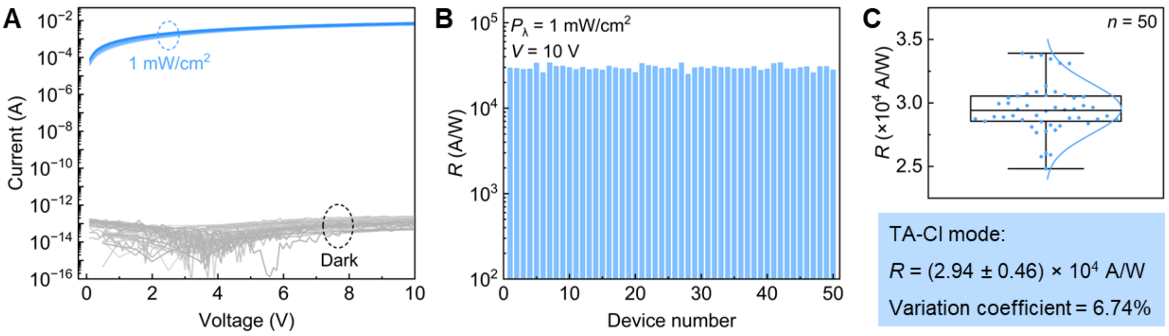

Fig. S17. Device-to-device variation of 50 devices operated in the trap-assisted charge injection (TA-CI) mode. (**A**) Current–voltage characteristics of 50 devices measured under dark and 365 nm illumination (1 mW/cm²). (**B**) Histogram of responsivity values measured at 10 V under 365 nm illumination (1 mW/cm²). (**C**) Box plot of responsivity distribution, yielding an average responsivity of (2.94 ± 0.46) × 10^4 A/W and a coefficient of variation of 6.74%.

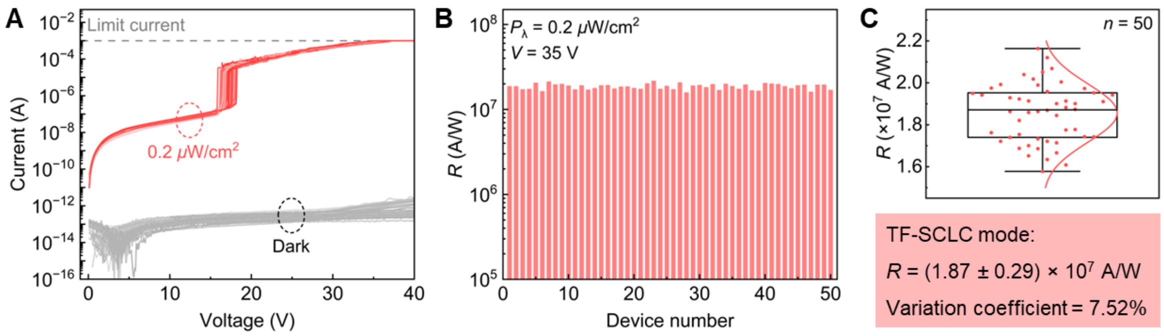

Fig. S18. Device-to-device variation of 50 devices operated in the trap-filled space-charge-limited current (TF-SCLC) mode. (**A**) Current–voltage characteristics of 50 devices measured under dark and 365 nm illumination (0.2 μW/cm²). (**B**) Histogram of responsivity values measured at 35 V under 365 nm illumination (0.2 μW/cm²). (**C**) Box plot of responsivity distribution, yielding an average responsivity of (1.87 ± 0.29) × 10^7 A/W and a coefficient of variation of 7.52%.

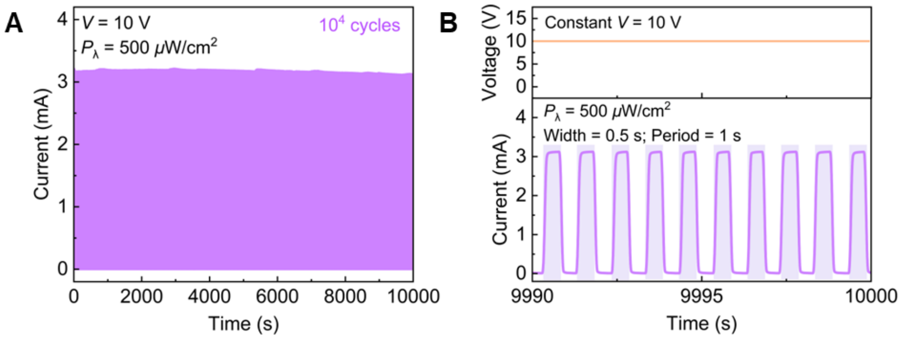

Fig. S19. Stability characteristics of the device operated in the trap-assisted charge injection (TA-CI) mode. (**A**) Stability characteristics of the device operated in the TA-CI mode after 10^4 light ON/OFF cycles. (**B**) Enlarged view of the stability curves, measured under a constant bias voltage of 10 V and 365 nm light pulse with a power intensity of 500 μW/cm^2, pulse width of 0.5 s, and period of 1 s.

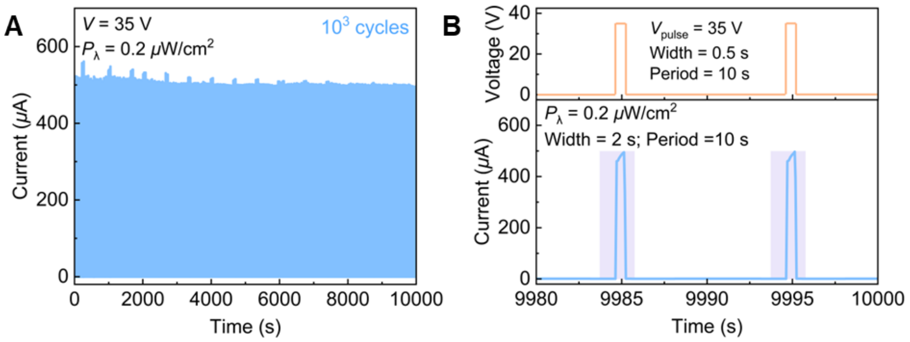

Fig. S20. Stability characteristics of the device operated in the trap-filled space-charge-limited current (TF-SCLC) mode. (**A**) Stability characteristics of the device operated in the TF-SCLC mode after 10^3 light ON/OFF cycles. (**B**) Enlarged view of the stability curves, measured under a 35 V bias pulse with a 0.5 s pulse width and a 10 s period, and a synchronized 365 nm light pulse with a power intensity of 0.2 μW/cm^2, pulse width of 2 s, and period of 10 s.

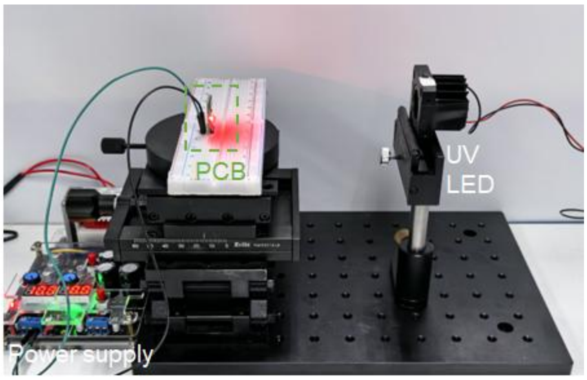

Fig. S21. The test platform of the UV visualization system. The test platform is on an optical platform, where the PCB under test is aligned with the UV LED.

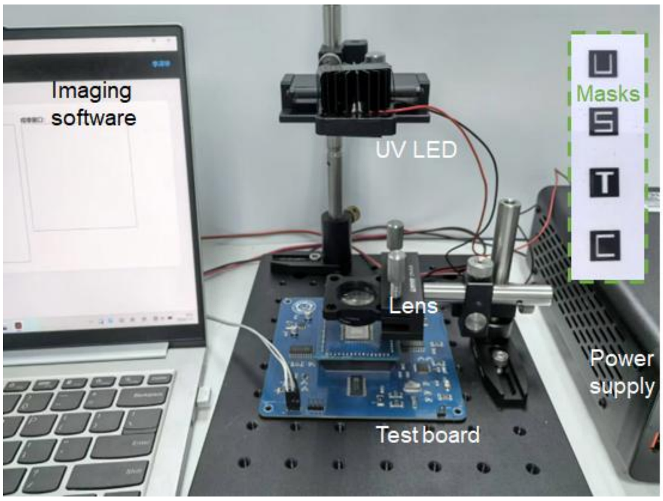

Fig. S22. Photograph of the whole imaging system.

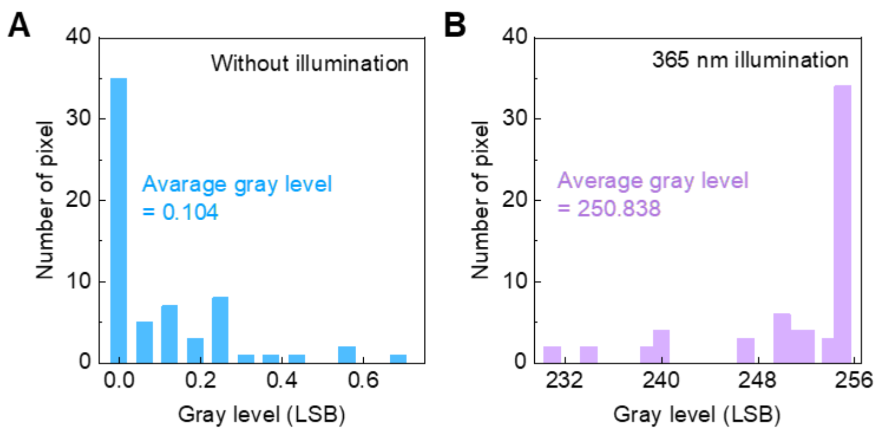

Fig. S23. Gray-level distributions of the photodetector array. (**A** and **B**) Gray-level distributions of all channels for the photodetector array (A) in the dark and (B) under uniform 365 nm light illumination.

Table S1. Performance comparison of a-GaON/GaN device with two-terminal photodetectors. Comparison of the key figure-of-merits between the a-GaON/GaN device and other two-terminal UV PDs based on wide-bandgap semiconductors, including GaN, ZnO, and SiC. The photoresponse properties (I_{dark}, D^*, *PDCR,* and R) of the a-GaON/GaN device in this work outperform those of most previously reported high-performance wide-bandgap photodetectors. ("N/A": not available; "—": not measured)

Material	I_{dark} (A)	*R* (A/W)	*PDCR*	*D** (Jones)	Light intensity	τ_r/τ_d (ms)	Ref.
**a-GaON/GaN**	**5.1×10^{-14}**	**38494**	**1.7×10^{10}**	**4.58×10^{18}**	**100 μW/cm^2**	**35/10**	**This work**
Zn-TPPOH/GaN	1×10^{-9}	4140	6.6×10^5	1.85×10^{14}	5 μW/cm^2	700/60	[39]
Ta_2NiSe_5/GaN	1×10^{-12}	12200	10^7	1.3×10^{16}	200 μW/cm^2	1.22/3.16	[40]
AlGaN/GaN	1×10^{-11}	7800	10^8	2.3×10^{15}	110 μW/cm^2	30/100	[41]
AlGaN/GaN	4×10^{-12}	20.9	10^6	1.9×10^{14}	1.96 μW/cm^2	180/900	[42]
$Mo_xRe_{1-x}S_2$/GaN	1×10^{-10}	888.69	1.48×10^6	6.1×10^{14}	11.9 mW/cm^2	181/259	[43]
Graphene/GaN	1.4×10^{-12}	100	4.8×10^7	1×10^{17}	95 μW/cm^2	0.345/0.364	[44]
ZnO	1×10^{-6}	181	1.4×10^3	4×10^{12}	N/A	N/A	[45]
ZnO	5×10^{-12}	400	10^6	3.7×10^{16}	400 μW/cm^2	6100/2100	[46]
$CsPbBr_3$/ZnO	5×10^{-14}	3500	10^7	6.6×10^{13}	2000 μW/cm^2	8/10	[47]
ZnO/ZnMgO	2.4×10^{-12}	8.92	1.02×10^7	1×10^{14}	N/A	N/A	[48]
ZnO/NiO	9.1×10^{-12}	6.9	8.9×10^4	8×10^{12}	1800 μW/cm^2	0.01/0.05	[49]
SiC/Graphene	5.2×10^{-14}	1.17	2×10^5	3.1×10^{14}	N/A	N/A	[50]
SnS_2/SiC	2×10^{-8}	22400	10^3	7.3×10^{13}	720 μW/cm^2	17/17	[51]
Graphene/SiC	5.9×10^{-9}	0.5645	1.7×10^6	5.4×10^{13}	N/A	0.013/—	[52]

Table S2. Performance comparison of a-GaON/GaN device with avalanche photodiodes and photomultiplier tubes. Comparison of the key figure-of-merits between the a-GaON/GaN device and other typically reported high-gain UV APDs based on wide-bandgap semiconductors (GaN, AlGaN, Ga_2O_3, and SiC) and commercial photomultiplier tubes (PMTs). ("N/A": not available)

**Material**	I_{dark} **(A)**	***R* (A/W)**	**Gain (a.u.)**	**λ (nm)**	**Light intensity**	**Bias (V)**	**Ref.**
**a-GaON/GaN**	7×10^{-13}	4.3×10^{7}	3.9×10^{6}	**365**	**0.1 μW/cm^2**	**35**	**This work**
GaN	8.1×10^{-5}	4223	4.1×10^{4}	360	1.82 mW/cm^2	111	[61]
GaN	1×10^{-4}	4.2×10^{5}	3×10^{6}	365	N/A	96.4	[62]
GaN	5.3×10^{-5}	3.8×10^{4}	1×10^{6}	355	N/A	70	[63]
AlGaN	1.2×10^{-3}	5.7×10^{5}	7×10^{6}	343	N/A	118.8	[18]
AlGaN	1×10^{-4}	1.9×10^{5}	1.3×10^{5}	324	N/A	79.5	[64]
AlGaN	3.1×10^{-7}	3.5×10^{4}	5.2×10^{5}	250	N/A	140	[65]
AlGaN	5×10^{-5}	3524	2×10^{4}	278	N/A	140	[66]
AlGaN	9.1×10^{-6}	103.5	2.3×10^{4}	340	N/A	66.5	[67]
AlGaN	5×10^{-4}	7.4×10^{4}	2×10^{6}	348	150 μW/cm^2	117	[68]
AlGaN	1×10^{-6}	6000	1×10^{5}	250	1 μW/cm^2	340	[69]
Ga_2O_3/MgO/ STO	1×10^{-9}	4.46×10^{5}	5.9×10^{5}	254	0.1 μW/cm^2	78.1	[9]
Ga_2O_3	1×10^{-10}	9780.23	1×10^{6}	254	64 μW/cm^2	60	[17]
Ga_2O_3/$BaSnO_3$	3×10^{-5}	1.5×10^{4}	1.6×10^{6}	254	84 μW/cm^2	46.5	[70]
ITO/Ga_2O_3	4×10^{-5}	5.9×10^{4}	6.8×10^{4}	254	10 μW/cm^2	40	[71]
Ga_2O_3/ZnO	5×10^{-6}	1.1×10^{4}	2.35×10^{5}	254	500 μW/cm^2	40	[72]
SiC	1×10^{-4}	1.56×10^{4}	1×10^{5}	282	N/A	157	[73]
SiC	3.17×10^{-4}	1×10^{4}	1×10^{5}	270	N/A	94	[74]
SiC	1×10^{-2}	1.8×10^{5}	1×10^{6}	274	N/A	156	[75]
SiC	1×10^{-7}	96.4	1000	270	N/A	260	[76]
PMT	$>1\times10^{-9}$	$>1.4\times10^{4}$	$>5\times10^{5}$	300-650	N/A	>1000	[77]
